# Distinguished Contributions in the Fields of Biomedical and Environmental Applications Incorporating Nanostructured Materials and Composites in Journal *Molecules*

**DOI:** 10.3390/molecules26082112

**Published:** 2021-04-07

**Authors:** Minas M. Stylianakis

**Affiliations:** Institute of Electronic Structure and Laser, Foundation for Research and Technology Hellas (FORTH), 70013 Heraklion, Crete, Greece; stylianakis@iesl.forth.gr

During the last two years, over 10,000 papers (articles, reviews, communications etc.) were published in *Molecules*. Most of them are excellent contributions that already centralized the readers’ and researchers’ interest, thus, gaining many citations; a fact that undoubtedly contributed to the journal’s impact factor boosting. In this frame, I feel extremely happy to write this editorial, recommending some high-quality published papers of the past biennium, aiming to provide a guide for researchers in various emerging fields of nanotechnology.

The extended use of nanostructured materials (2D and carbon-based, metal, and metal oxide nanoparticles, etc.), significantly improved the performance as well as the lifespan of every day and more specialized applications, such as optoelectronics [1], energy production and storage [2], and other biomedical and environmental applications [2]. In addition to combination with polymers and small molecules, biomaterials, advanced the formation of composites exhibiting improved mechanical, optical, electrical, antimicrobial, sensing, and catalytic properties [3].

In this context, the scope of this editorial is to highlight some excellent studies and review articles related to the incorporation of nanostructured materials and composites in biomedical, sensing, and environmental applications, including water treatment technologies and the development of environment-friendly coatings, as briefly displayed in Figure 1. Therefore, six research articles and six reviews are demonstrated and discussed below, aiming to inspire the research community, towards the development or improvement of multifunctional nanostructured composite materials for relevant purposes.

The first part concerns the incorporation of nanocomposites in biomedical applications and consists of three research and three review articles. More specifically, Ai et al. [4] developed a composite film with exceptional hydrophilicity, swellability, stability, and mechanical properties, comprised of silk sericin (SS), polyvinyl alcohol (PVA), zinc oxide (ZnO) nanoparticles (NPs), and polydopamine (PDA). The advanced composite film also exhibited excellent antibacterial properties against *Escherichia coli* and *Staphylococcus aureus*, indicating its great potential towards the realization of wound dressings. In a similar context, Li et al. [5] prepared biodegradable wound dressing fibers using a gelatin mat that embedded ZnO/graphene oxide (GO) nanocomposites and showed enhanced antibacterial properties against the same above-mentioned bacteria. In another work, Grande-Tovar et al. [6] investigated the potential applicability of various chitosan (CS)/glutaraldehyde (GLA)-based composites incorporating titanium dioxide NPs (TiO_2_) and GO, in cell regeneration and biomedical applications. Biocompatibility and long-term stability of the nanocomposites were assessed upon subdermal implantation in Wistar rat tissues.

Bei et al. [7] presented a comprehensive mini review on the beneficial role of graphene-based nanocomposites for neural tissue engineering and compared their properties with that of other emerging materials. The limitations of graphene-based composites are also discussed in terms of the impact on the differentiation and regeneration of neural stem cells. In addition, Xing et al. [8] attempted to predict future trends in the field of food applications, providing a summary of excellent studies on the preparation and properties of edible materials for edible coatings and films (ECF) containing antimicrobial NPs. All possible antimicrobial mechanisms that might occur, as well as the physical, mechanical, and releasing characteristics of ECF that correlated to the concentration of the embedded NPs, are discussed in terms of suitability for fruit and vegetable storage. The last review article related to biomedical applications by Oprea et al. [9] recapitulates the findings from several reports on nanocellulose metal oxides hybrid composites formation, properties, and applications. It was deduced that the combination of nanocellulose and metal oxides, depending on the synthesis method and conditions might impact the thermal and mechanical properties of the hybrid composites, along with their bactericidal and cytotoxicity response.

In the topic of sensing applications, Wang et al. [10] demonstrated their excellent study on the development and evaluation of ZnO/graphene oxide (GO) composite sensing materials for acetone vapor detection. Due to the dual role of GO, as both the template and sensitizer, as well as the synergistic effects between ZnO and GO, the composites displayed remarkable sensitivity, selectivity, and response/recovery times. On other hand, Petrucci et al. [11] provided a summary of the most recent studies on the detection of natural methylxanthines such as caffeine, theophylline, and theobromine, using GO and reduced graphene oxide (RGO), as starting materials. In this context, the role of GO and RGO as adsorbents and sensor components, as well as their toxicity in case of biomedical use were discussed in detail.

Some excellent contributions in the field of environmental applications are included in the last part of this editorial. Soares et al. [12] explored the use of trimethyl chitosan/siloxane coated Fe_3_O_4_ (Fe_3_O_4_@SiO_2_/SiTMC) core@shell magnetic NPs as nanosorbents for the antibiotic sulfamethoxazole (SMX) removal from water. The prepared core@shell NPs presented great potential to capture SMX from aqueous solutions, combining high adsorption performance and great magnetic properties, contrary to other reported sorbents. In addition, a very informative review article by Jani et al. [13] included current studies in the field of water treatment technology related to zero-dimensional (0D) carbon allotropes/polymer nanocomposite membranes. The preparation methods and the extraordinary properties of the composite membranes ascribed to the presence of 0D carbon-based materials for water and wastewater treatment applications were analyzed, while current and future challenges and opportunities are discussed.

A brilliant research article by Kong et al. [14] who studied the incorporation of biomass derived nanocellulose in a polyurethane (PU)-based paint and the effect on its mechanical properties is also suggested. The prepared composite paint exhibited enhanced tensile strength, elongation at break, hardness and abrasion resistance, compared to the pristine PU, thus, meeting the criteria to serve as a promising waterborne wood coating. In this context, it was pointed out that a broader exploitation of biomass materials could take place, aimed at the development of environment-friendly and scalable processing of PU-based paints.

My last suggestion is a broad review article by Zhu et al. [15] that summed up the most recent advances in the use of nanostructured MoO_3_-based materials in the fields of energy and environmental catalysis. In this overview, the crystalline structure and properties, as well as the current challenges and opportunities of using MoO_3_ and its composites in such energy applications were demonstrated and analyzed.

To conclude, I highly recommend these outstanding studies and comprehensive review articles in the fields of biomedicine and environment applications incorporating nanostructures. I consider many details in terms of synthesis, processing, characterization, challenges, and potential of such nanocomposites, which is a very informative collection for early stage and experienced researchers, working in these fields.

## Figures and Tables

**Figure 1:**
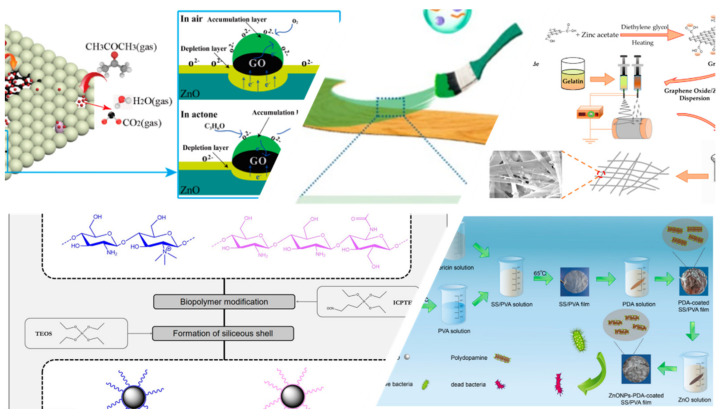
An overview of the editorial’s contents.

## Data Availability

No new data were created or analyzed in this study. Data sharing is not applicable to this article.
